# Hotels

**Published:** 1859-03

**Authors:** 


### HOTELS.

A new feature is inaugurated in hotel life, and greatly needed too. The proprietor of the Astor House, New York, advertises that, among other desireable things in his worldfamed hotel, "invalids will be especially attended to." If that should be so, it is the only hotel in New York in which "special attention" is *not* given to the special neglect of the unfortunate sick.

